# Supplementary material for: BAM15‐mediated mitochondrial uncoupling protects against obesity and improves glycemic control
Source: EMBO Mol Med. 2020 Jun 10;12(7):e12088. doi: 10.15252/emmm.202012088 (PMC7338798; doi:10.15252/emmm.202012088)
Supplement: Supplementary file 1 — Appendix [file EMMM-12-e12088-s001.pdf]

APPENDIX

BAM15 Mediated Mitochondrial Uncoupling Protects Against Obesity and Improves Glycemic Control

Christopher L. Axelrod, William T. King, Gangarao Davuluri, Robert C. Noland, Jacob Hall, Michaela Hull, Wagner S. Dantas, Elizabeth R.M. Zunica, Stephanie J. Alexopoulos, Kyle L. Hoehn, Ingeborg Langohr, Krisztian Stadler, Haylee Doyle, Eva Schmidt, Stephan Nieuwoudt, Kelly Fitzgerald, Kathryn Pergola, Hisashi Fujioka, Jacob T. Mey, Ciaran Fealy, Anny Mulya, Robbie Beyl, Charles L. Hoppel, and John P. Kirwan

<u>Table of Contents</u>

Appendix Figure S1. Related to Figure 7. Increased skeletal muscle fatty acid oxidation is supported by AMPK mediated suppression of lipogenesis in white adipose tissue following treatment with BAM15.

Appendix Table S1. Related to Figures 1 and 7. qRT-PCR primer sequences.

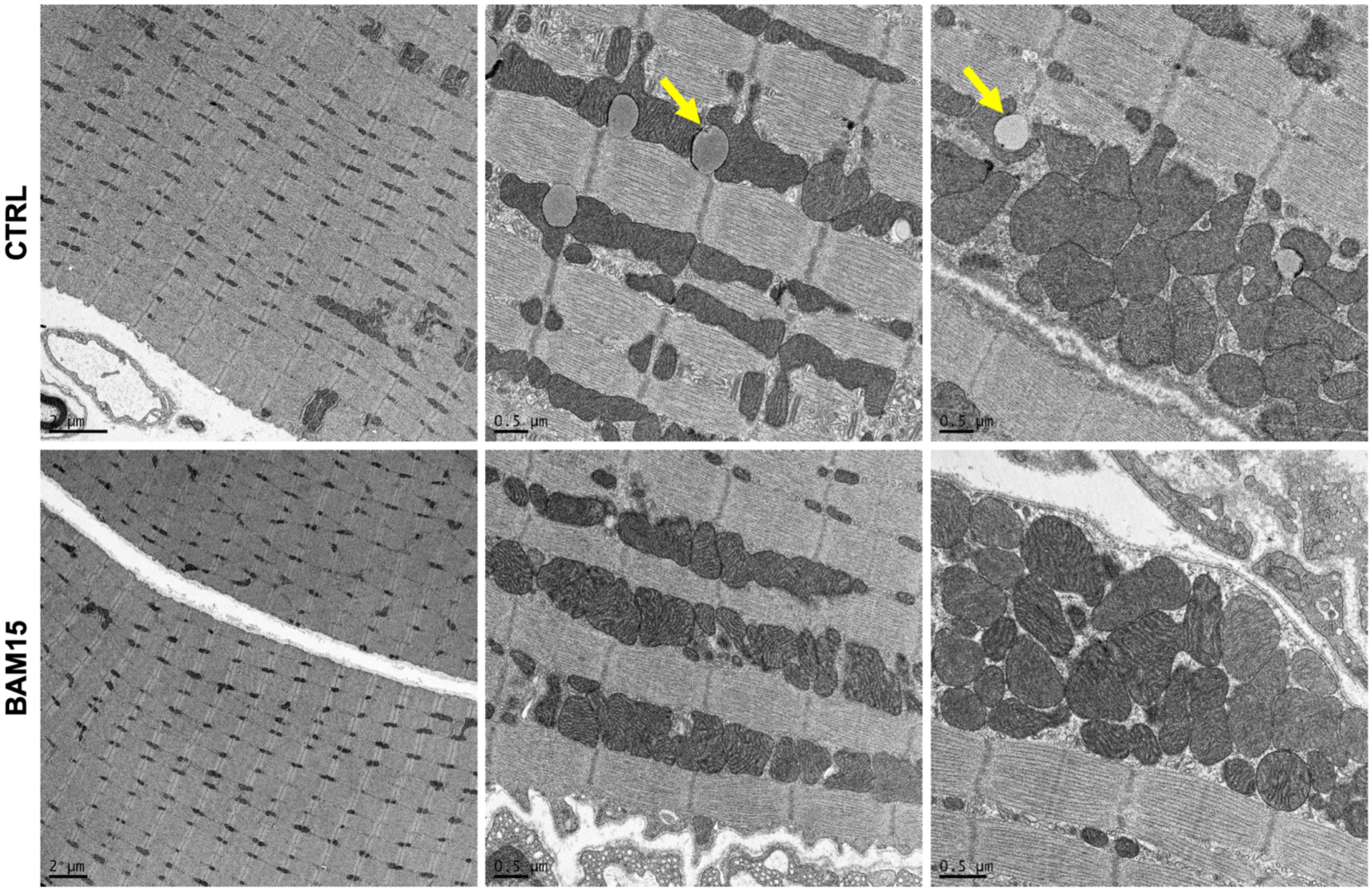
CTRL
BAM15
2 μm
0.5 μm
0.5 μm
2 μm
0.5 μm
0.5 μm

Appendix Figure S1.

Representative transmission electron microscopy (TEM) from of phosphate-buffered aldehyde-DMSO immersed tissue sections. Left: 2 μm sections of predominantly white gastrocnemius muscle. Center: 0.5 μm sections of intermyofibrillar mitochondria from red gastrocnemius muscle. Right: 0.5 μm sections of subsarcolemmal mitochondria from red gastrocnemius muscle. Yellow arrow denotes identification of a lipid droplet.

Appendix Table S1. qRT-PCR primer sequences.

Probe	5'→3' Forward Primer	5'→3' Reverse Primer
Pparg	GTACTGTCGGTTTCAGAAGTGCC	ATCTCCGCCAACAGCTTCTCCT
Lpl	CCA ATG GAG GCA CTT TCC A	CCACGTCTCCGAGTCCTCTCT
Pnpla2	TGTGGCCTCATTCCTCCTAC	TCGTGGATGTTGGTGGAGCT
Lipe	GGAGCACTACAAACGCAACGA	TCGGCCACCGGTAAAGAG
Acaca	CTGGCTGCATCCATTATGTCA	TGGTAGACTGCCCGTGTGAA
Acacb	AGAAGCGAGCACTGCAAGGTTG	GGAAGATGGACTCCACCTGGTT
Scd1	GCAAGCTCTACACCTGCCTCT T	CGTGCCTTGTAAGTTCTGTGGC
Mlxipl	GAGTGCTTGAGCCTG GCTTACA	GCTCTCCAGATGGCGTTGTTCA
Srebf1	CGACTACATCCGCTTCTTGCAG	CCTCCATAGACACATCTGTGCC
Fasn	TCCTGGAACGAGAACACGATCT	GAGACGTGTCACTCCTGGACTTG
18s	TAGAGGGACAAGTGGCGTTC	CGCTGAGCCAGTCAGTGT
Cox2	GCCGACTAAATCAAGCAACA	CAATGGGCATAAAGCTATGG
