# Supplementary material for: BAM15‐mediated mitochondrial uncoupling protects against obesity and improves glycemic control
Source: EMBO Mol Med. 2020 Jun 10;12(7):e12088. doi: 10.15252/emmm.202012088 (PMC7338798; doi:10.15252/emmm.202012088)
Supplement: Supplementary file 2 — Expanded View Figures PDF [file EMMM-12-e12088-s002.pdf]

Expanded View Figures

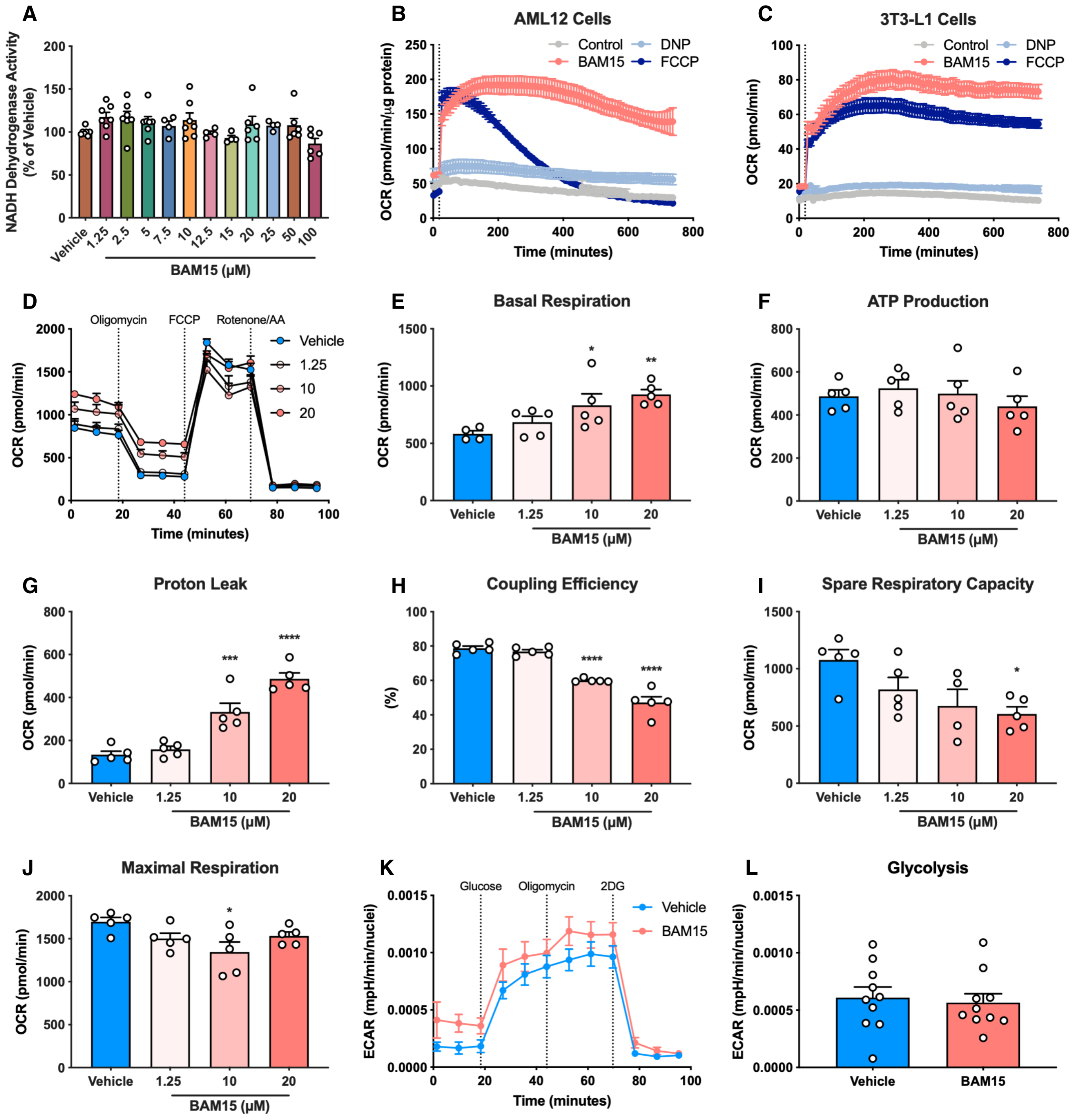

Figure EV1.

Figure EV1. Related to Fig 1. BAM15 improves cellular respiratory kinetics by sustained mitochondrial uncoupling.

A NADH dehydrogenase activity in C2C12 cells following 16-h treatment with varying concentrations of BAM15 (μM) (*N* = 7 per condition).

B, C (B) Mitochondrial respiratory kinetics following acute injection of 1 μM BAM15, FCCP, or DNP into AML12 hepatocytes and (C) 3T3-L1 adipocytes (*N* = 5 per condition).

D–J Assessment of intact oxygen consumption rates (OCR) in C2C12 cells after serial injection of oligomycin, FCCP, and a cocktail of rotenone and antimycin A following overnight incubation with 0 (vehicle; 0.01% DMSO), 1.25, 10, or 20 μM BAM15 (*N* = 5 per condition). Basal: vehicle versus 10 μM: $P = 0.037$, vehicle versus 20 μM: $P = 0.0072$. Proton leak: vehicle versus 10 μM: $P = 0.0002$, vehicle versus 20 μM: $P < 0.0001$. Coupling efficiency: vehicle versus 10 μM: $P < 0.0001$, vehicle versus 20 μM: $P < 0.0001$. Spare respiratory capacity: vehicle versus 20 μM: $P = 0.0179$. Maximal respiration: vehicle versus 10 μM: $P = 0.0192$.

K, L Assessment of intact extracellular acidification rates (ECAR) in C2C12 cells after serial injection of glucose, oligomycin, and 2-deoxyglucose after 16-h treatment with 0 (vehicle; 0.01% DMSO) or 20 μM BAM15 (*N* = 10 per condition).

Data information: Data are shown as the mean ± SEM. *$P < 0.05$, ***$P < 0.01$, ****$P < 0.001$. Comparisons of treatment × time were assessed by one-way repeated-measures ANOVA with Tukey's multiple comparisons. Comparisons of treatment alone were assessed by an unpaired Student's *t*-test.

© 2020 The Authors

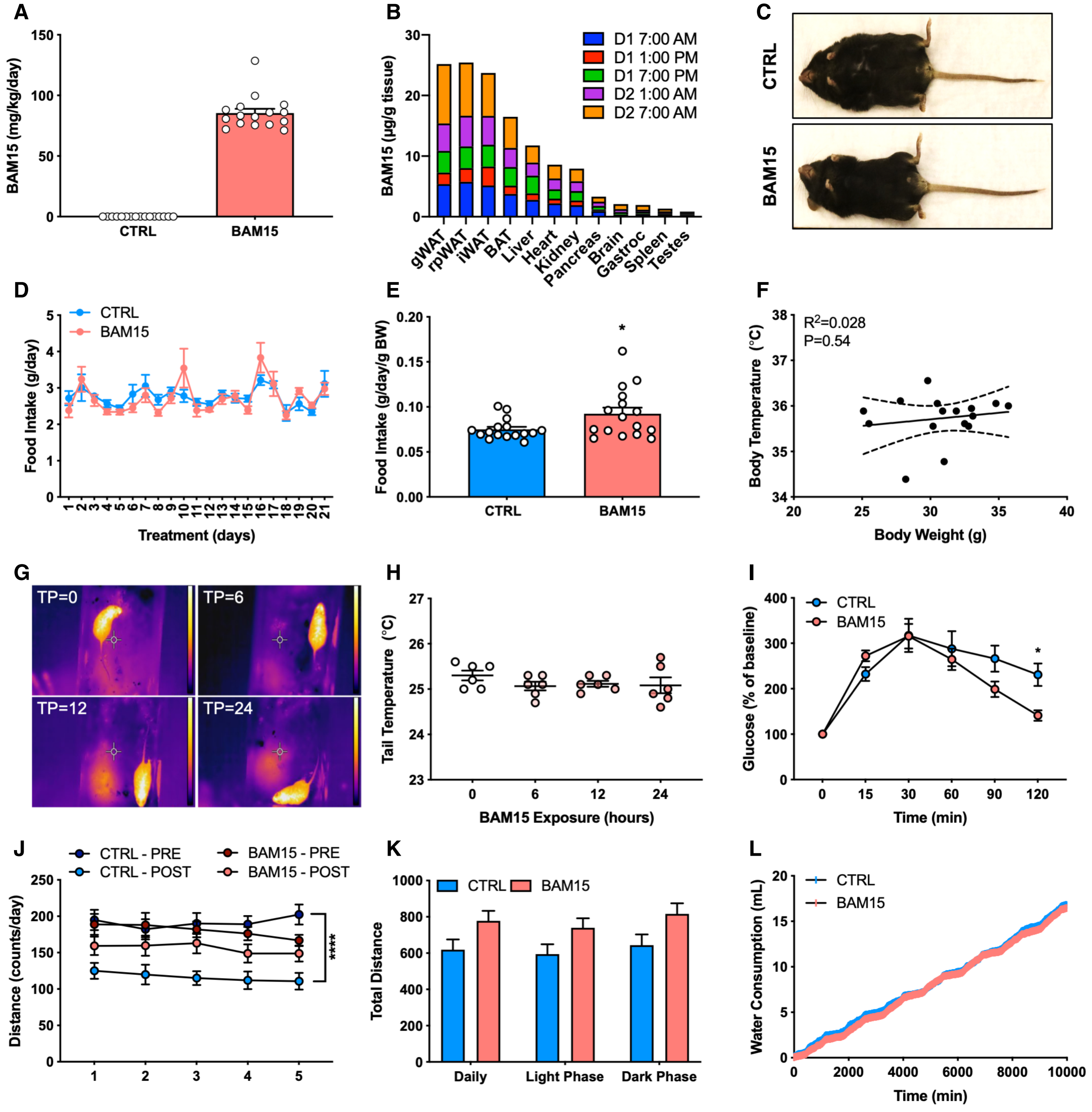

Figure EV2.

© 2020 The Authors EMBO Molecular Medicine e12088 | 2020 23 of 20

Figure EV2. Related to Fig 4. BAM15 prevents diet-induced obesity by increasing energy expenditure in C57BL/6J mice.

A Representative image of a CTRL- and BAM15-treated animal at time of necropsy.

B, C (B) Daily consumption (*N* = 16 per group) and (C) tissue distribution of BAM15 over a 24-h feeding period (*N* = 3 per time point).

D, E (D) Daily food intake over the 3-week treatment period (*N* = 16 per group) and (E) mean food intake expressed relative to body weight (*N* = 16 per group; *P* = 0.0275).

F Relationship between body temperature and weight after 3 weeks of treatment (*N* = 16).

G, H (G) Representative thermal image of a mouse exposed to 0, 6, 12, and 24 h of BAM15 (TP = 0, 6, 12, and 24, respectively). Scale bar (right of image) represents 22–32° (dark blue to white) temperature range. (H) Tail heat dissipation of mice exposed to 0, 6, 12, and 24 h of BAM15 (*N* = 6 per time point).

I Glucose concentrations in response to IPGTT expressed as a percent of baseline (*N* = 8 per group; 120 min: *P* = 0.0413).

J–L (J) Daily physical activity before (PRE) and 3 weeks (POST) of CTRL or BAM15 treatment (*N* = 8 per group; CTRL PRE–POST: *P* < 0.0001) and (K) cumulative physical activity following 3 weeks of CTRL or BAM15 treatment (*N* = 8 per group). (L) Cumulative water consumption over a 7-day period ((*N* = 8 per group).

Data information: Data are shown as the mean ± SEM. *$P < 0.05$, ****$P < 0.001$. Comparisons of treatment × time were assessed by two-way repeated-measures ANOVA with Tukey's multiple comparisons. Panels J-L were assessed by a repeated-measures ANOVA with body weight as a covariate. Comparisons of treatment alone were assessed by an unpaired Student's *t*-test. Bivariate relationships were determined by linear regression modeling followed by statistical evaluation with Pearson's r. Dotted lines represent 95% confidence intervals.

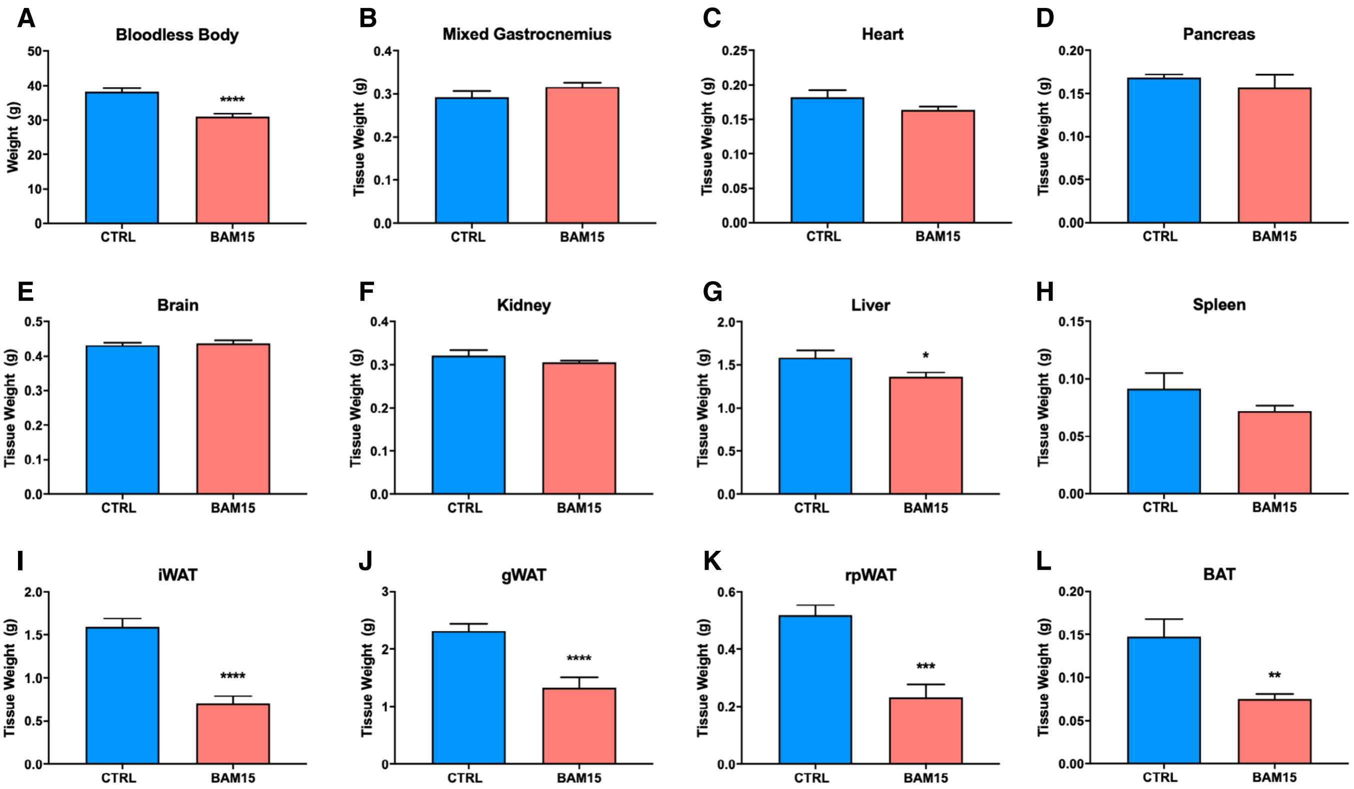

Figure EV3. Related to Fig 4. BAM15 prevents diet-induced obesity by increasing energy expenditure in C57BL/6J mice.

A–L Organ weights at time of necropsy following 3 weeks of CTRL or BAM15 treatment (*N* = 8 per group). Data are shown as the mean ± SEM. *$P < 0.05$, **$P < 0.01$, ***$P < 0.01$, ****$P < 0.001$. Comparisons of treatment alone were assessed by an unpaired Student's *t*-test. Bloodless body: $P < 0.0001$. Liver: $P = 0.0257$. iWAT: $P < 0.0001$. gWAT: $P < 0.0001$. rpWAT: $P = 0.0002$. BAT: $P = 0.0064$.

© 2020 The Authors

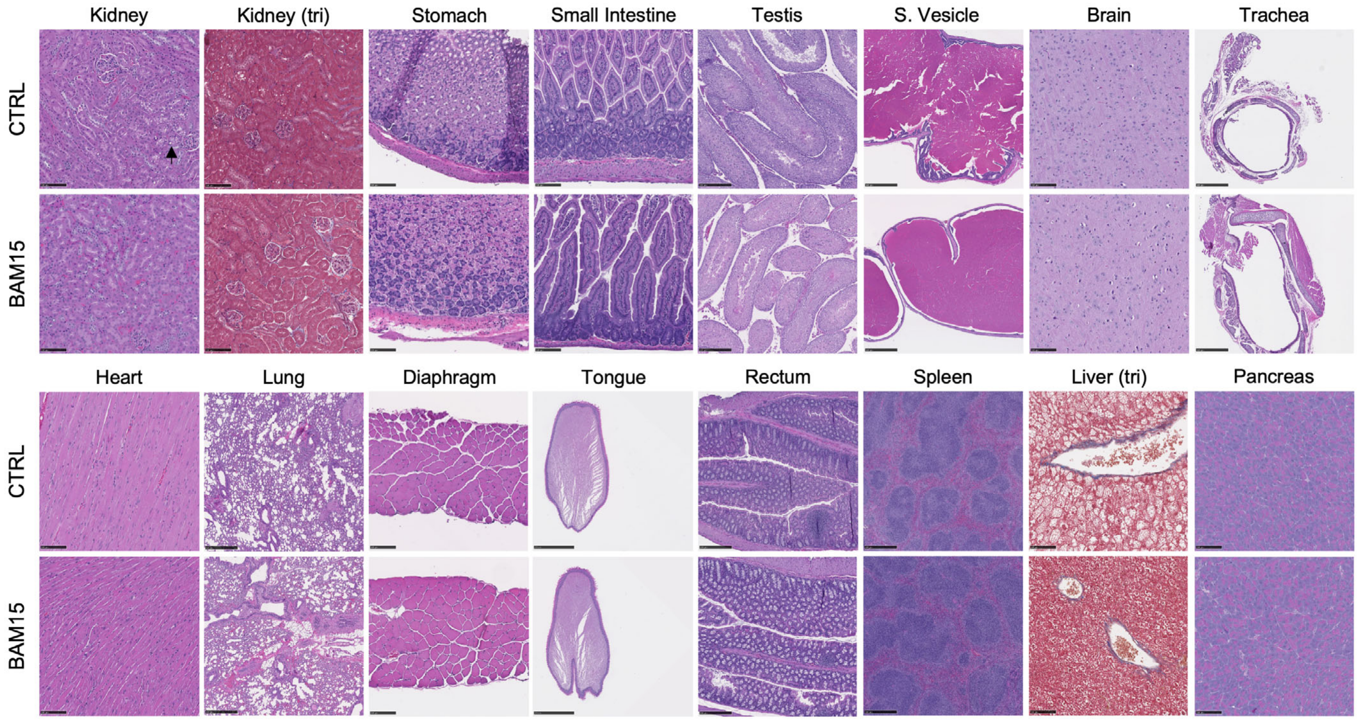

Figure EV4. Related to Fig 6. BAM15 restricts adipose tissue expansion and lipid deposition to liver and kidney in C57BL/6J mice. **Related to Fig 6. BAM15 restricts adipose tissue expansion and lipid deposition to liver and kidney in C57BL/6J mice.**

Representative H&E and trichrome staining of paraffin-embedded tissue sections. Scale bars (black) = 100 µm with exception to testis (250 µm), seminal vesicles (500 µm), trachea (500 µm), lung (500 µm), tongue (2.5 mm), rectum (250 µm), and spleen (500 µm). Black arrow represents vacuole formation in the proximal tubules of the kidney. Representative H&E and trichrome staining of paraffin-embedded tissue sections. Scale bars (black) = 100 µm with exception to testis (250 µm), seminal vesicles (500 µm), trachea (500 µm), lung (500 µm), tongue (2.5 mm), rectum (250 µm), and spleen (500 µm). Black arrow represents vacuole formation in the proximal tubules of the kidney.

© 2020 The Authors

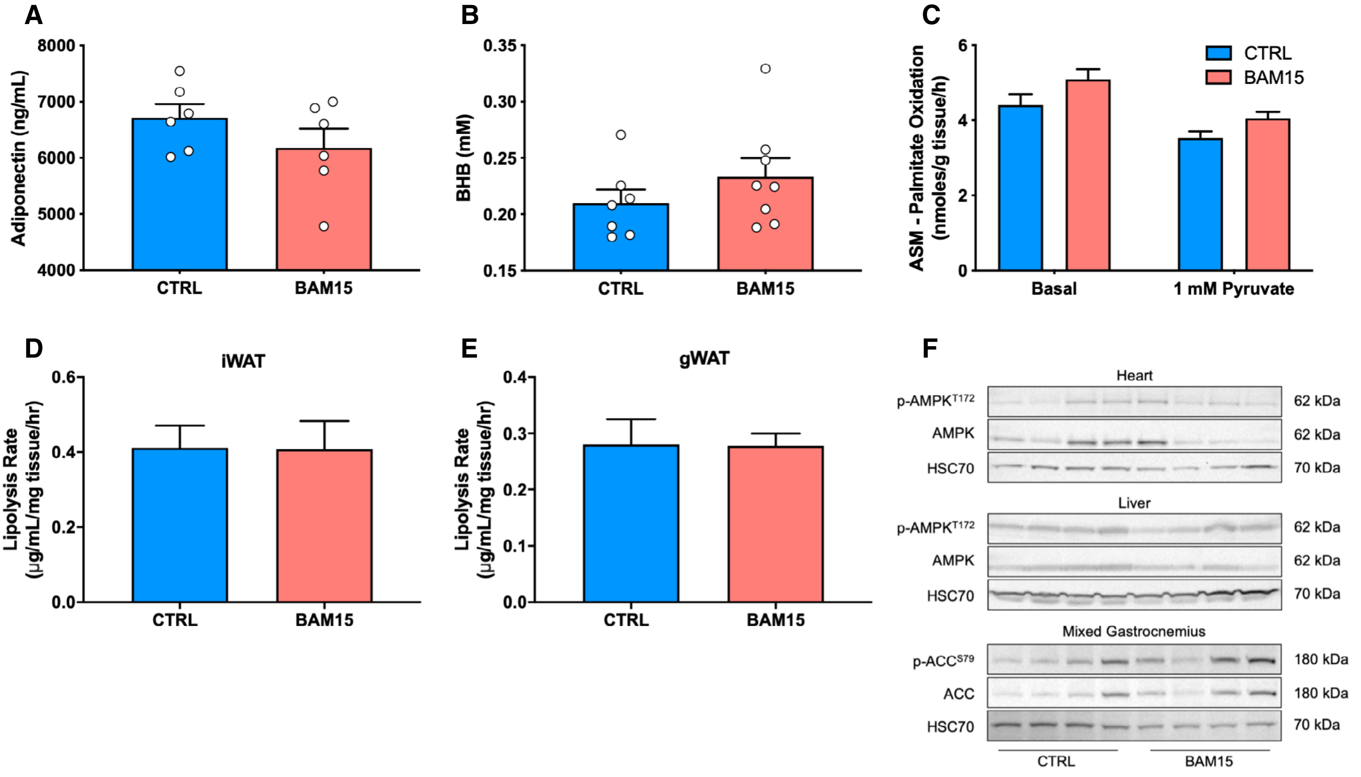

Figure EV5. Related to Fig 6. BAM15 restricts adipose tissue expansion and lipid deposition to liver and kidney in C57BL/6J mice.

A, B (A) Circulating adiponectin ($N = 6$ per group) and (B) beta-hydroxybutyrate (BHB) (CTRL $N = 7$, BAM15 $N = 8$) concentrations in CTRL- and BAM15-treated animals.

C Fatty acid oxidation attributable to acid-soluble metabolites (ASM) in mixed gastrocnemius muscle ± 1 mM pyruvate ($N = 7$ per group).

D, E (D) *Ex vivo* rates of glycerol release from iWAT and (E) gWAT in CTRAL- and BAM15-treated animals ($N = 8$ per group).

F Representative immunoblots of p-AMPK^{T172} and AMPK from heart and liver homogenates and p-ACC^{S79} and ACC from mixed gastrocnemius muscle.

Data information: Data are shown as the mean ± SEM. Main effects of treatment × time were assessed by two-way repeated-measures ANOVA with Tukey's post hoc test. Main effects of treatment alone were assessed by unpaired Student's *t*-test.

Source data are available online for this figure.

© 2020 The Authors
