# Supplementary figures and images for: BAM15‐mediated mitochondrial uncoupling protects against obesity and improves glycemic control
Source: EMBO Mol Med. 2020 Jun 10;12(7):e12088. doi: 10.15252/emmm.202012088 (PMC7338798; doi:10.15252/emmm.202012088)

# Source Data - Figure EV5

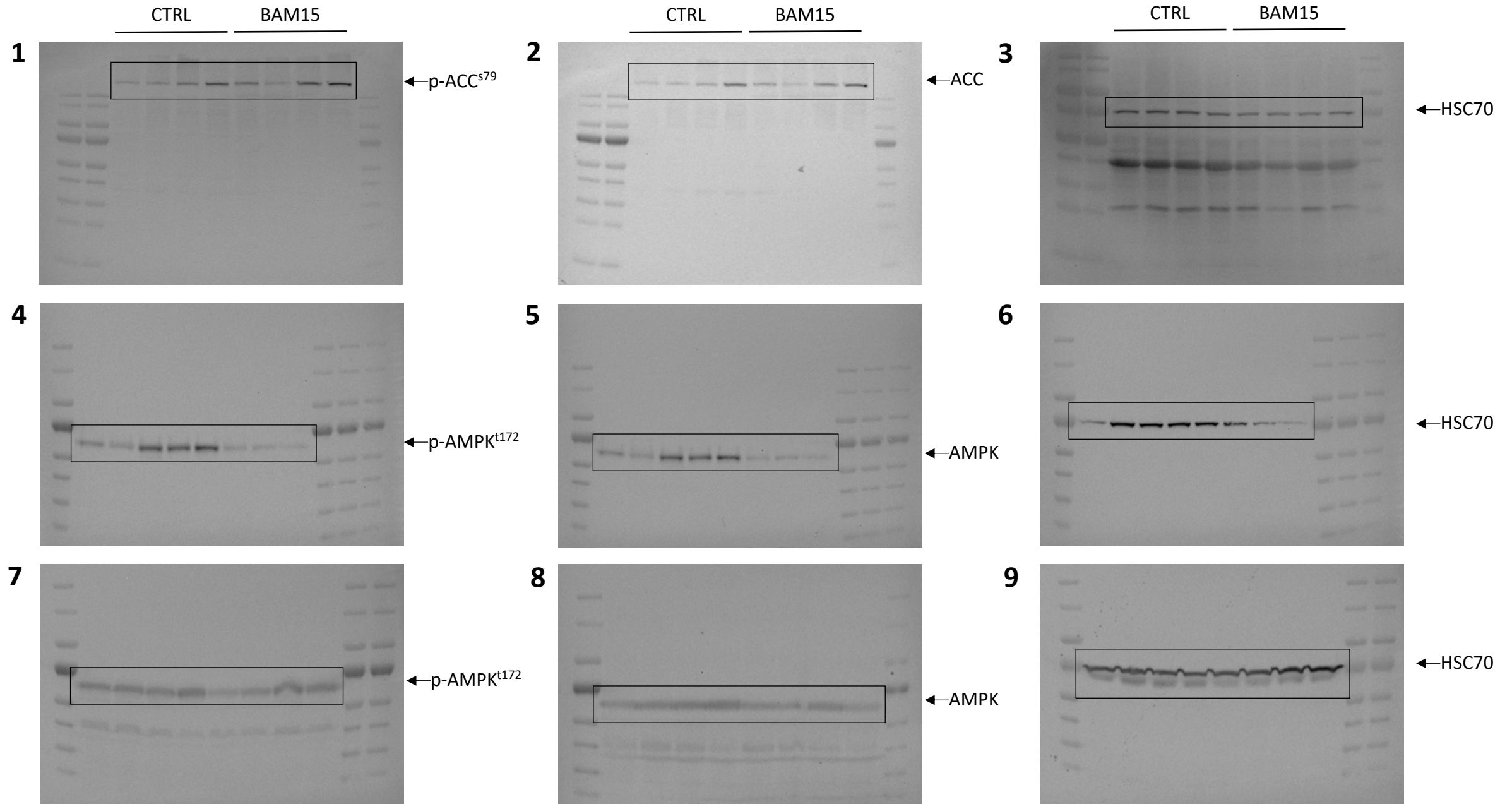

Supplement: Supplementary file 3 — Source Data for Expanded View [file EMMM-12-e12088-s005.zip › emmm202012088-sup-0005-SDataFig5EV.pdf]

# Source Data - Figure 7H

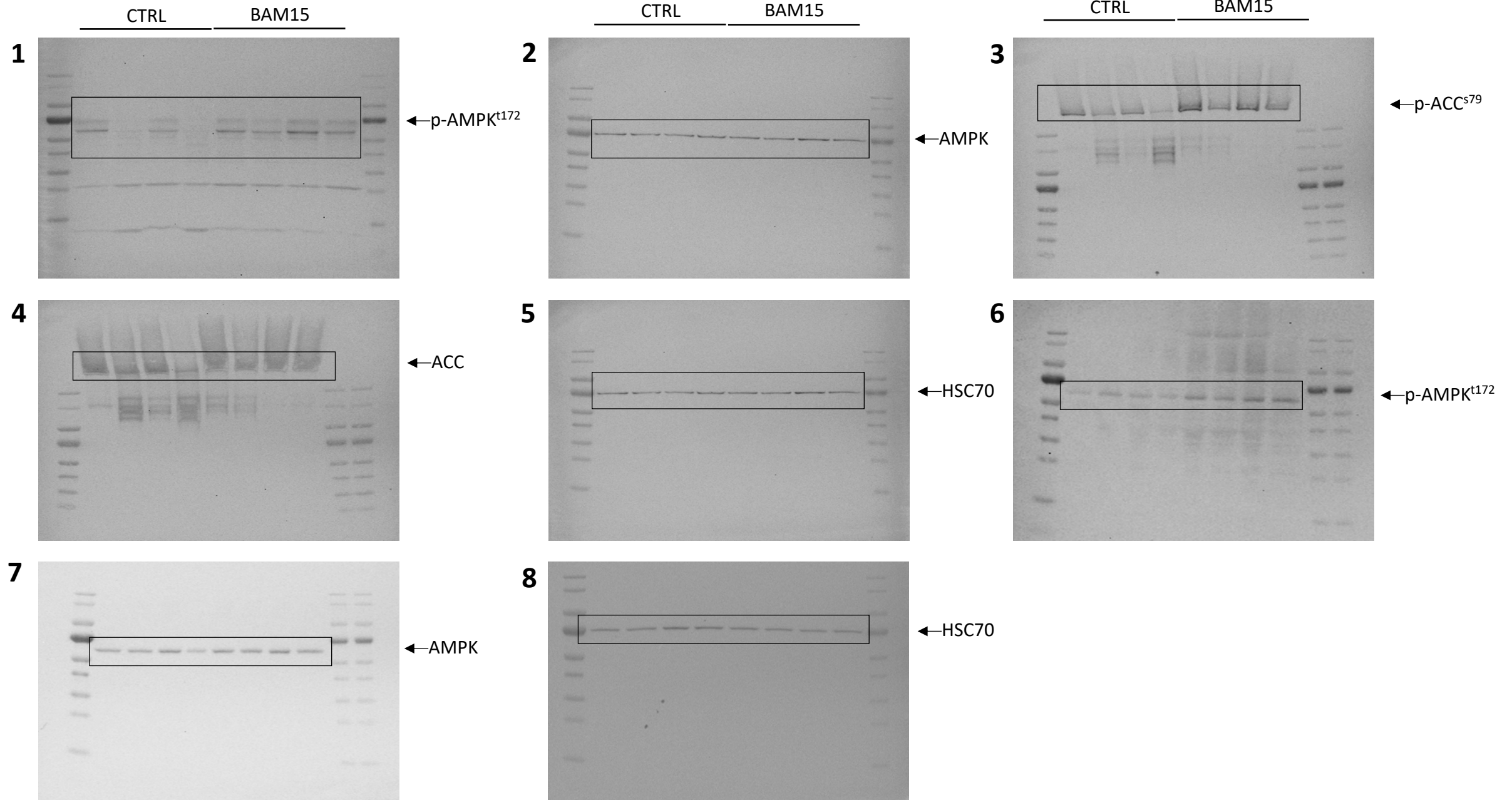

Supplement: Supplementary file 6 — Source Data for Figure 7 [file EMMM-12-e12088-s004.pdf]
