# Supplementary material for: BAM15‐mediated mitochondrial uncoupling protects against obesity and improves glycemic control
Source: EMBO Mol Med. 2020 Jun 10;12(7):e12088. doi: 10.15252/emmm.202012088 (PMC7338798; doi:10.15252/emmm.202012088)

Source Data - Figure 3A

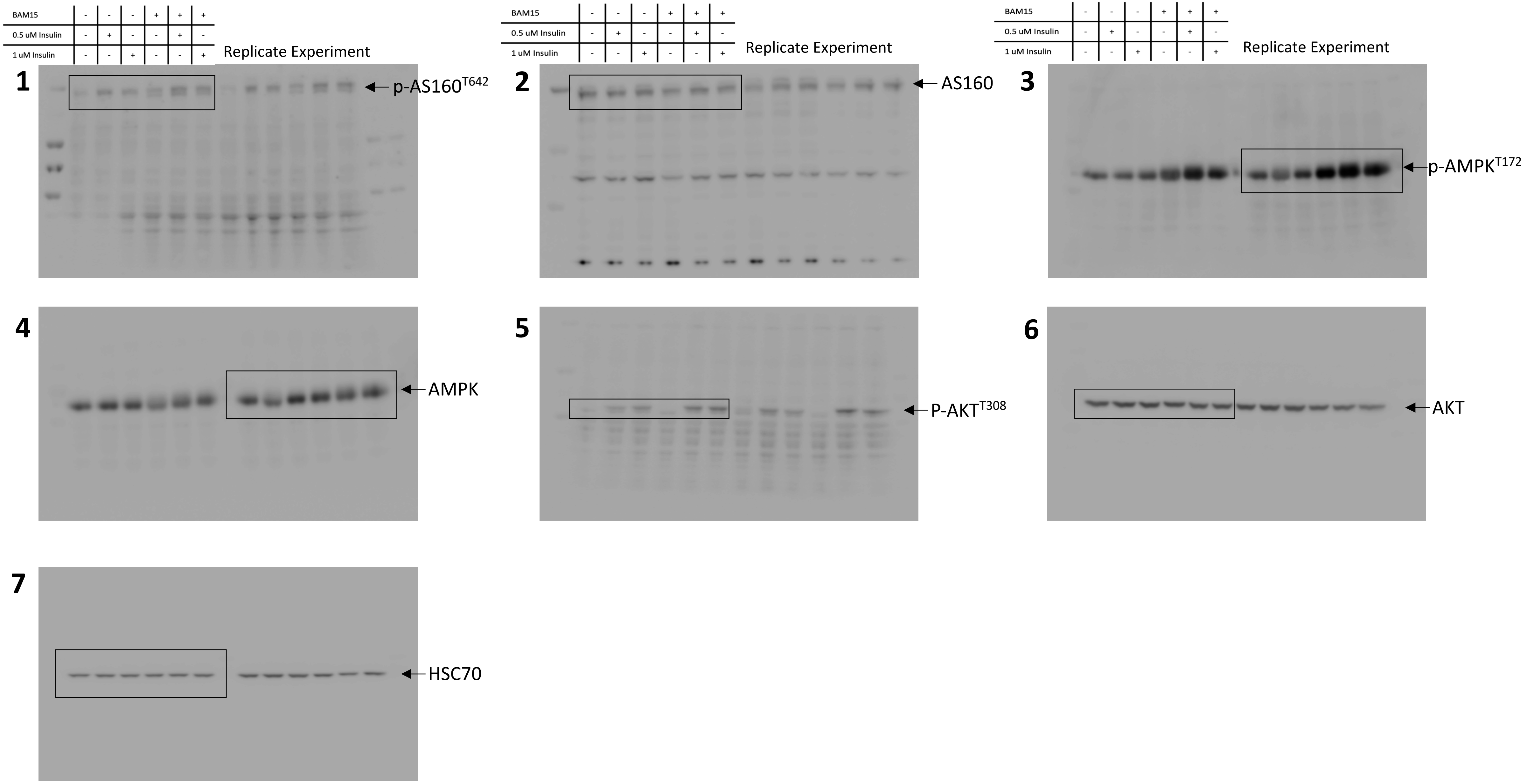

Source Data - Figure 3E

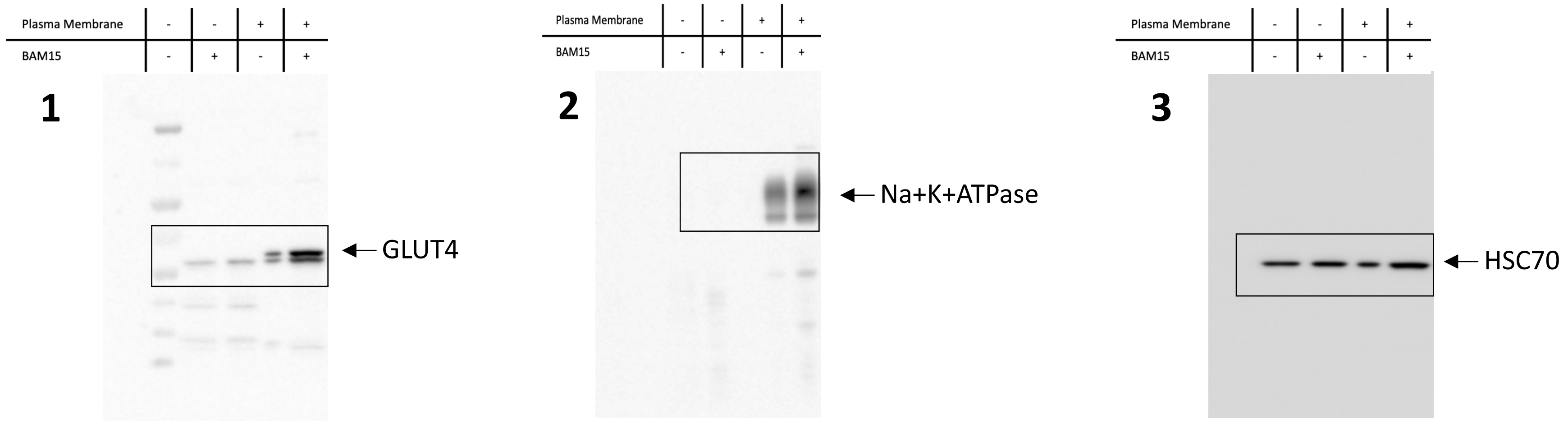

Source Data - Figure 3I

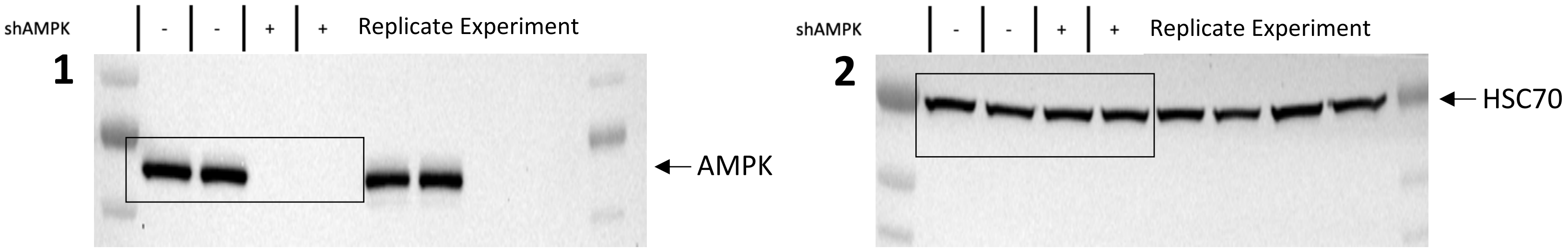

Source Data - Figure 3M

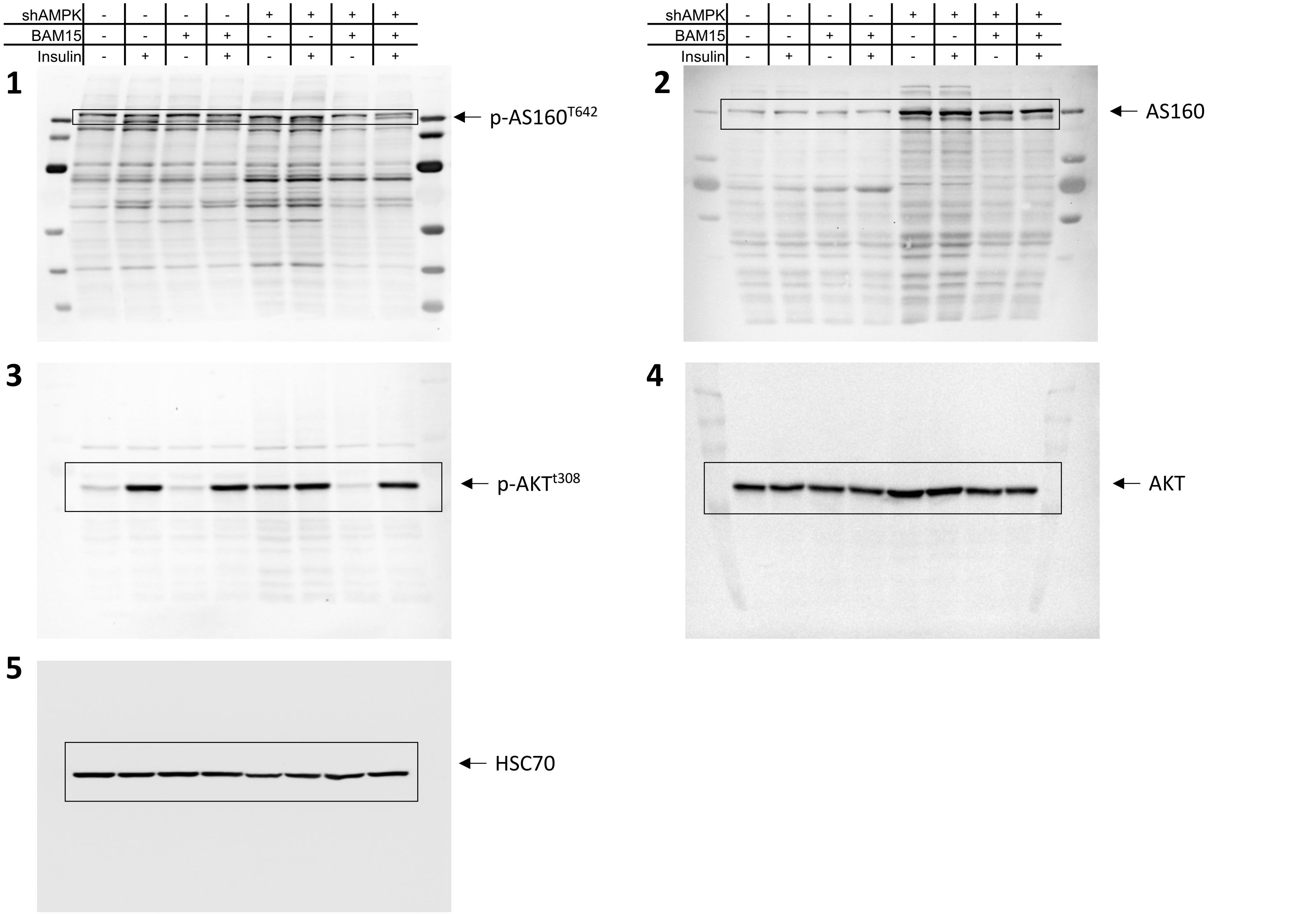

Supplement: Supplementary file 5 — Source Data for Figure 3 [file EMMM-12-e12088-s003.pdf]
